# Gene Regulatory Identification Based on the Novel Hybrid Time-Delayed Method

**DOI:** 10.3389/fgene.2022.888786

**Published:** 2022-05-19

**Authors:** Wenzheng Bao, Xiao Lin, Bin Yang, Baitong Chen

**Affiliations:** ^1^ School of Information Engineering, Xuzhou University of Technology, Xuzhou, China; ^2^ Department of Pharmaceutics, Zaozhuang Municipal Hospital, Zaozhuang, China; ^3^ School of Information Science and Engineering, Zaozhuang University, Zaozhuang, China 277160; ^4^ Xuzhou Municipal First People’s Hospital, Xuzhou, China

**Keywords:** gene regulatory network, time-delayed, complex-valued, S-system model, system biology

## Abstract

Gene regulatory network (GRN) inference with biology data is a difficult and serious issue in the field of system biology. In order to detect the direct associations of GRN more accurately, a novel two-step GRN inference technique based on the time-delayed correlation coefficient (TDCC) and time-delayed complex-valued S-system model (TDCVSS) is proposed. First, a TDCC algorithm is utilized to construct an initial network. Second, a TDCVSS model is utilized to prune the network topology in order to delete false-positive regulatory relationships for each target gene. The complex-valued restricted additive tree and complex-valued differential evolution are proposed to approximate the optimal TDCVSS model. Finally, the overall network could be inferred by integrating the regulations of all target genes. Two real gene expression datasets from *E. coli* and *S. cerevisiae* gene networks are utilized to evaluate the performances of our proposed two-step GRN inference algorithm. The results demonstrated that the proposed algorithm could infer GRN more correct than classical methods and time-delayed methods.

## Introduction

With the accomplishment of the human genome project and the emergence of high-throughput gene analysis techniques, it has been recognized that great heterogeneity in gene mutation profiles of cancer tissues has been reported (Thomas et al., 2007; Tesniere et al., 2010; Rupaimoole et al., 2016; Zhang et al., 2018). Mutant genes are not only numerous and complex but could also construct a dynamic network system. At the cell level, interrelated genes/proteins constitute complex cellular networks, including signaling pathways, gene regulatory networks (GRN), and metabolic networks (Quach et al., 2007; Ma’ayan, 2009). When it comes to the GRN issue, any gene could not execute the independent function. They must coordinate with other genes to complete certain biological processes and participate in determining the behaviors and phenotypes of cells (Hernández- Prieto et al., 2014). Thus, the research on GRN has become a hotspot in the field of system biology during the past several decades (Bracken et al., 2016; Liu et al., 2018).

The gene regulatory network contains two parts, including nodes and edges. The main work is to identify the direct regulations of all pairs of nodes. In real organisms, genetic and non-genetic factors could cause an objective phenomenon that time delays occur in the gene expression process. Recently, several machine learning methods have been utilized to identify time-delayed regulations among genes (Parmar et al., 2015; Wang et al., 2020). Lo proposed a causal network model by identifying causal-directed regulations with time delays (Lo et al., 2015). Kordmahalleh et al. presented a hierarchical recurrent neural network (HRNN) and genetic algorithm (GA) to infer the time-delayed gene regulatory network (Kordmahalleh et al., 2017). Li et al. utilized the Max–Min high-order dynamic Bayesian network (MMHO-DBN) to identify the synthetic and real time–delayed gene regulatory network (TDGRN) (Hu et al., 2020). Yu and Li also utilized the dynamic Bayesian network (DBN) to infer the TDGEN (Li et al., 2014; Yu et al., 2017). Some time-delayed information theory methods have been proposed to infer the TDGEN, such as TimeDelay-ARACNE (Zoppoli et al., 2010) and the time-lagged correlation method (Sefidmazgi et al., 2016; Abduallah and Wang, 2017). Zhao et al. proposed a kind of delay differential equation model to infer the gene regulatory networks (Zhao et al., 2016).

The S-system model is a nonlinear ordinary differential equation (ODE) composed of power-law functions, which is very suitable for simulating nonlinear biological systems with a large number of components, such as GRNs and metabolic pathways (Miyawaki-Kuwakado et al., 2020). Liu et al. proposed a multiobjective optimization method to evolve the S-system in order to infer the biochemical network (Liu and Wang, 2008). Orland et al. utilized simulated annealing (SA) to search the optimal parameters of S-system in order to simulate real biochemical networks (Gonzalez et al., 2007). Wang et al. proposed a simplified S-system and a multi-dimensional optimization method for GRN inference (Wang et al., 2010). Iwata et al. utilized the S-system to simulate metabolic reaction systems (Iwata et al., 2014). Chowdhury et al. proposed the time-delayed S-system and stochastic S-system to model time-delayed and stochastic regulations in GRN, respectively, and gained a good performance (Chowdhury et al., 2013a; Chowdhury et al., 2013b; Ji et al., 2017).

With several decades of efforts, complex-valued methods have been proposed to solve the real prediction and classification issues. Compared with real-valued methods, complex-valued methods have stronger modeling and noise tolerance abilities (Yang and Bao, 2019; Yuan et al., 2021). Fink et al. proposed a complex-valued multilayer feedforward neural network to forecast the degradation of railway track turnouts (Fink et al., 2014). Chen et al. utilized a complex-valued radial basis function network to solve a nonlinear signal processing problem (Chen et al., 1994). Goh et al. utilized complex-valued recurrent neural networks (RNNs) to predict Santa Fe and chaotic Mackey–Glass time series data (Goh et al., 2006). Savitha et al. proposed a complex-valued version of the extreme learning machine to solve real-valued classification problems (Savitha et al., 2012). Rashid utilized the complex-valued neural network to solve classification problems in the bioinformatics field (Rashid et al., 2016). Bakbak et al. presented the complex wavelet neural network to classify the sonar signal (Bakbak and Peker, 2020).

In order to enhance the accuracy of GRN inference, this study presents a time-delayed complex-valued S-system model (TDCVSS) to identify time-delayed and nonlinear relationships among genes. Compared with the S-system, TDCVSS contains time-delayed and complex-valued parameters, and the variables are complex-valued. The time-delayed correlation coefficient (TDCC) algorithm is first utilized to construct a TDCC matrix and the optimal time delay vector between genes. According to the TDCC matrix, the initial network is constructed. A complex-valued hybrid swarm intelligent algorithm based on the restricted additive tree and differential evolution is utilized to search for the optimal TDCVSS model in order to prune the network topology further.

## Methods

### Time-Delayed Correlation Coefficient

The time-delayed correlation coefficient (TDCC) is utilized to evaluate the linear relationship between two genes under the conditions of a constant system time delay, which is described as follows.

$$R_{X,Y}\left(\tau \right)=\frac{\sum_{t=1}^{T}\left(X\left(t\right)-\bar{X}\right)\left(Y\left(t+\tau \right)-\bar{Y}\right)}{\sqrt{\sum_{t=1}^{T}{\left(X\left(t\right)-\bar{X}\right)}^{2}\sum_{t=1}^{T}{\left(Y\left(t+\tau \right)-\bar{Y}\right)}^{2}}},$$ (1)

where X(t) and Y(t+τ) are gene expression profiles of gene X and gene Y at the time points t and t+τ , respectively. T is the number of sample points, and $\bar{X}$ and $\bar{Y}$ are the means of gene expression levels of gene X and gene Y , respectively.

### Time-Delayed Complex-Valued Time-Delayed S-System

The time-delayed complex-valued S-system (TDCVSS) is the time-delayed and complex-valued version of the S-system. Compared with a real-valued S-system, TDCVSS contains two improvements. Input variables $\left(X_{1},X_{2},\ldots X_{N}\right)$ and rate constants ( $\alpha_{i}$ and $\beta_{i}$ ) are complex-valued. Also, in a TDCVSS model, a time-delayed factor (τ) is included. The i−th time-delayed and complex-valued differential equation is given in Eq. 2.

$${X^{\prime }}_{i}\left(t\right)=\alpha_{i}\prod_{j=1}^{N}X_{j}^{g_{ij}}\left(t+\tau_{ij}\right)-\beta_{i}\prod_{j=1}^{N}X_{j}^{h_{ij}}\left(t+\tau_{ij}\right),$$ (2)

where $h_{ij}$ and $g_{ij}$ are real-valued kinetic orders. $\tau_{ij}$ is the time delay between variable $X_{i}$ and variable $X_{j}$ .

### Complex-Valued Restricted Additive Tree

**FIGURE 1:**
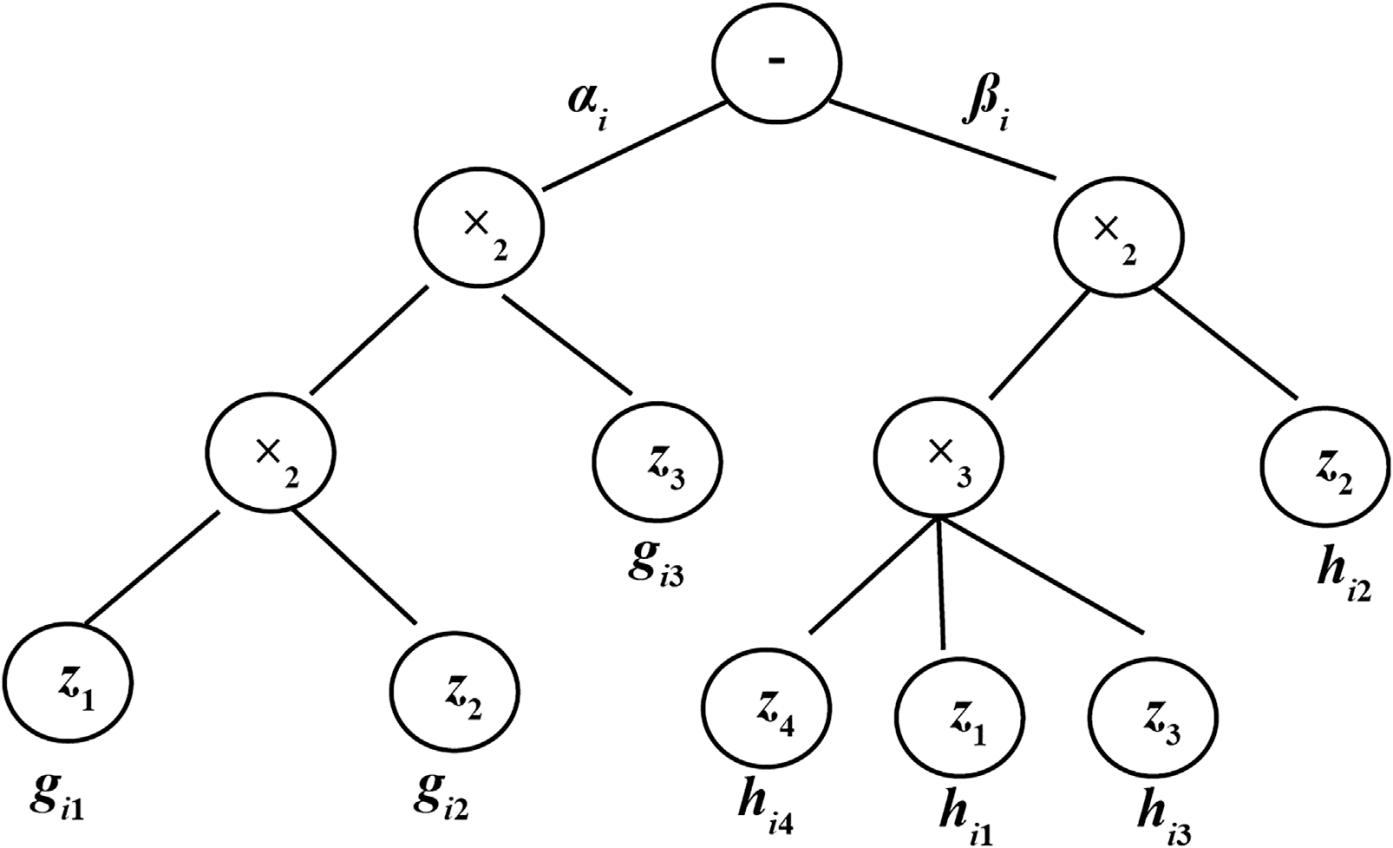
Example of the chromosome of CVRAT.

The TDCVSS model contains complex-valued variables and coefficients. In the GRN, each target gene corresponds to a small number of regulatory factors. Thus, for each dependent variable in TDCVSS, the proper independent variables need to be selected. A complex-valued restricted additive tree algorithm (CVRAT) is utilized to evolve the structure of the model. An example of the chromosome of TDCVSS can be demonstrated in Figure 1. The node in the first layer is fixed to subtraction (-). Two operator sets ( $F=\left\{X_{2},X_{3},\ldots ,X_{n}\right\}$ and $V=\left\{z_{1},z_{2},\ldots ,z_{n}\right\}$ ) were utilized to create the nodes randomly in other layers. $X_{i}$ is the product of i complex-valued input variables. In order to represent the parameters of the TDCVSS model, a real-valued parameter ( $h_{ij}$ or $g_{ij}$ ) is given to each variable node and a complex-valued parameter ( $\alpha_{i}$ or $\beta_{i}$ ) is given to each branch of the root node. The time delay vector between variable i and other variables is given as $\left[\tau_{i1},\tau_{i2},\ldots ,\tau_{iN}\right]$ . The corresponding TDCVSS model is $\frac{dz_{i}}{dt}=\alpha_{i}z_{1}^{g_{i1}}\left(t+\tau_{i1}\right)z_{2}^{g_{i2}}\left(t+\tau_{i2}\right)z_{3}^{g_{i3}}\left(t+\tau_{i3}\right)-\beta_{i}z_{1}^{h_{i1}}\left(t+\tau_{i1}\right)z_{2}^{h_{i2}}\left(t+\tau_{i2}\right)z_{3}^{h_{i3}}\left(t+\tau_{i3}\right)z_{4}^{h_{i4}}\left(t+\tau_{i4}\right)$ .

**FIGURE 2:**
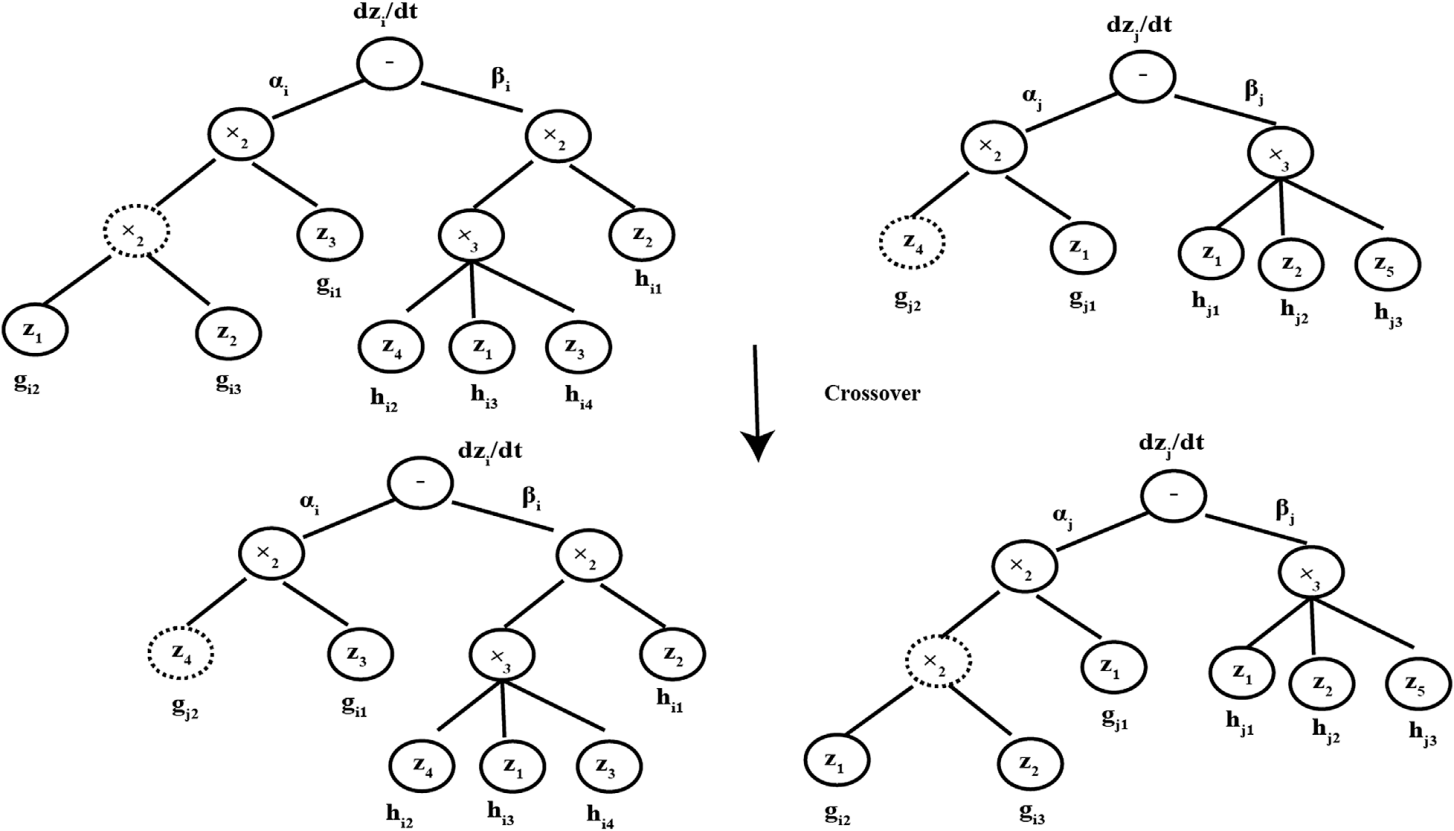
Crossover operator.

**FIGURE 3:**
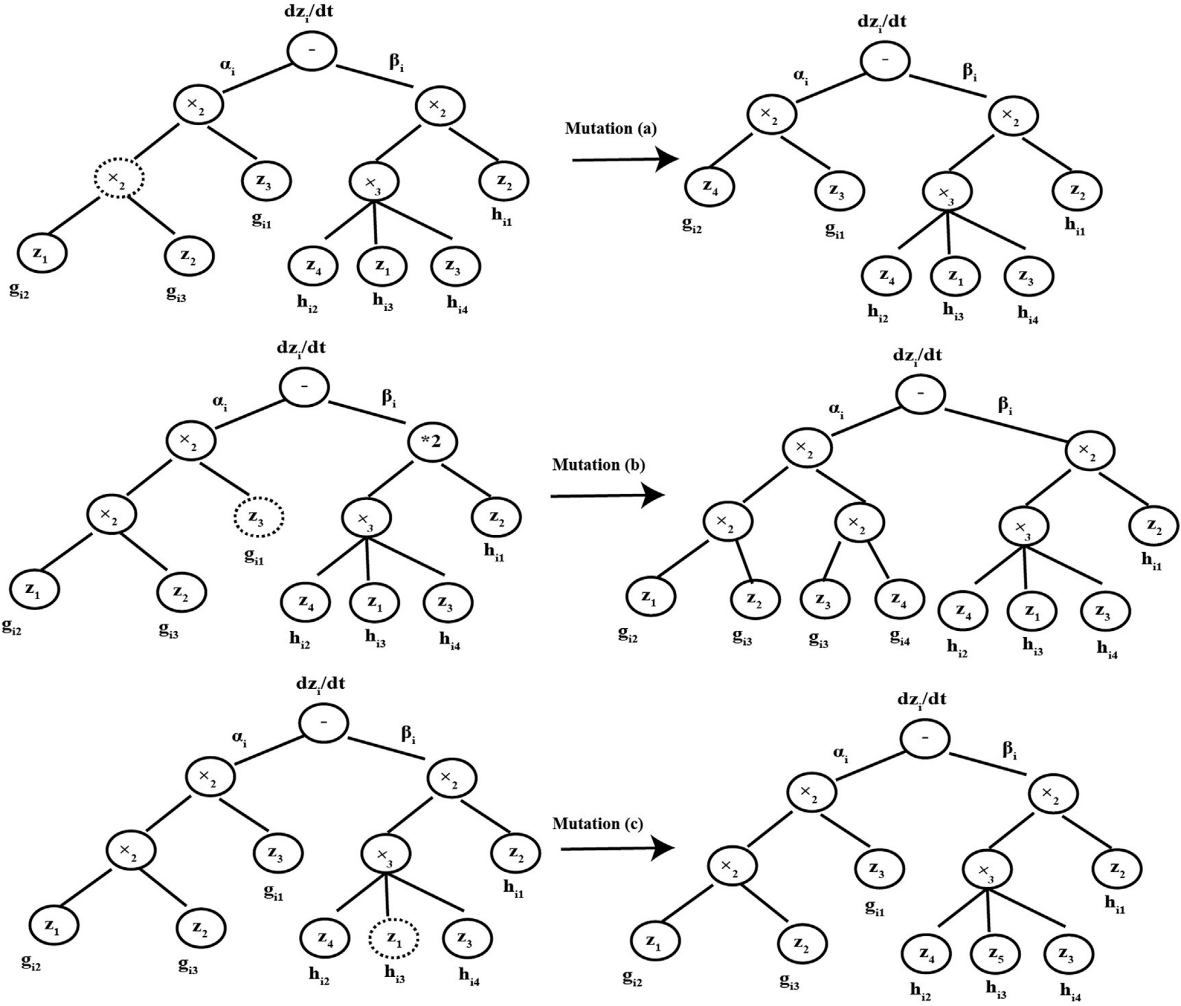
Mutation operators.

In the CVRAT algorithm, three genetic operators (selection, crossover, and mutation) are used to evolve the chromosome populations, which are the same as some structure-based evolutionary algorithms, such as genetic programming (GP). The detail crossover and mutation operators are shown in Figures 2, 3, respectively (Yang et al., 2020).

### Complex-Valued Differential Evolution

Differential evolution (DE) is an efficient and global evolutionary algorithm, which is based on the continuous variable optimization (Das and Suganthan, 2011). Its idea comes from the genetic algorithm (GA), which also contains the crossover, mutation, and reproduction. However, the mutation vector of differential evolution is generated by the difference vector of the parent generation, and the new individual could be generated by the crossover of the parent individuals. Considering its simple structure, easy implementation, and strong robustness, DE is widely utilized in many fields, such as bioinformatics, image processing, document extraction, artificial neural network, and electromagnetics. Complex-valued differential evolution (CVDE) is the complex-valued version of DE. In CVDE, a complex-valued individual includes the real part and the imaginary part, which need to be evolved simultaneously. CVDE could improve the diversity of population and premature convergence of DE. The optimization process of parameters of TDCVSS with CVDE is introduced in Algorithm 1.

Algorithm 1 Parameter optimization of TDCVSS with complex-valued differential evolution.

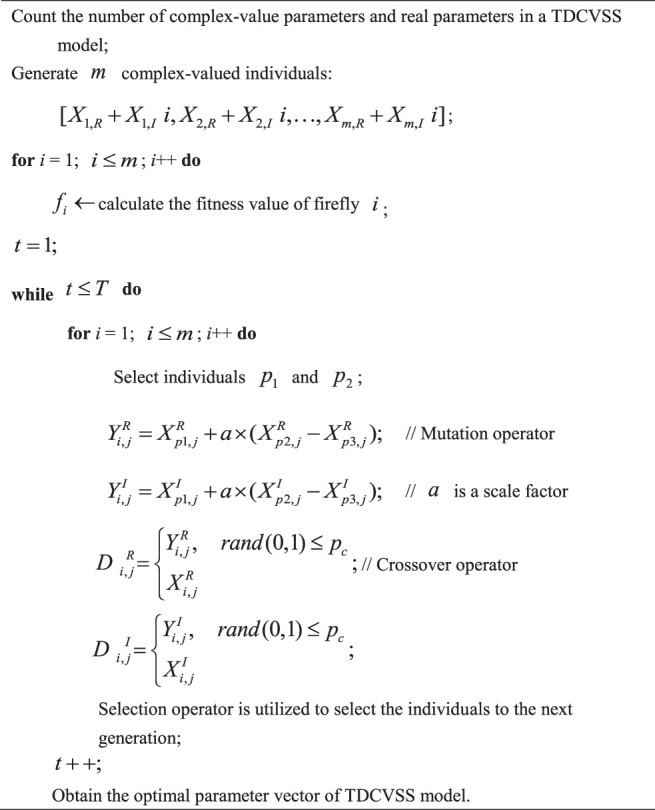

### Gene Regulatory Network Inference Algorithm

**FIGURE 4:**
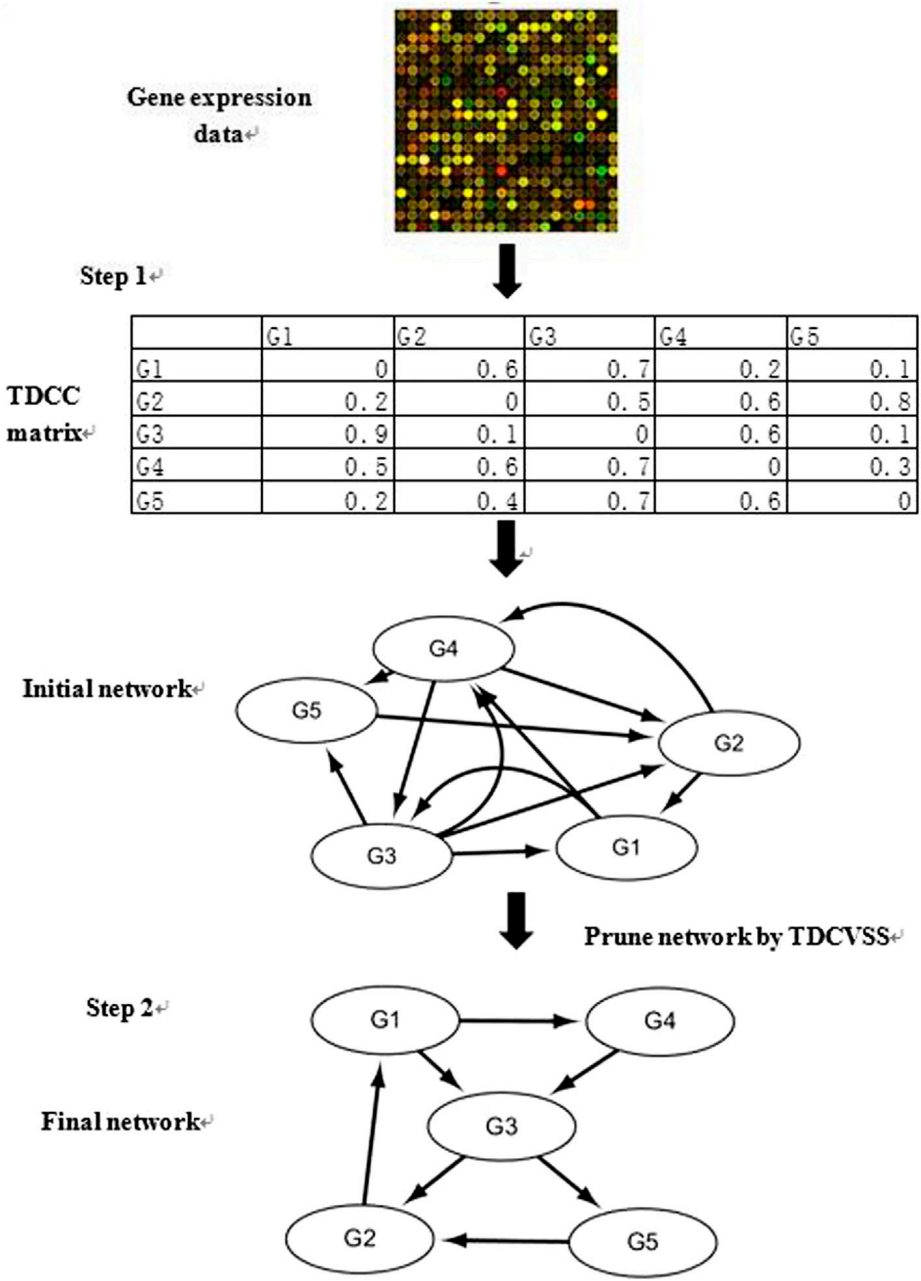
Flowchart of gene regulatory network inference.

The proposed network inference algorithm contains two steps, whose flowchart is demonstrated in Figure 4.

Step 1 Construction of the Initial Gene Regulatory Network

1) According to the given gene expression data, the TDCC method is utilized to obtain the TDCC matrix and the optimal time delay vector between regulatory factors and target genes. The maximum time delay $T_{\max}$ is set, and the CC values are calculated among genes with the different time delays. The maximum CC value and time delay are selected as TDCC and the optimal time-delayed value between the regulatory factor and target gene separately.
2) According to the TDCC matrix, a directed weighted graph is obtained. The initial network is constructed with the selected threshold. If the weight of an edge is less than the threshold, the edge is deleted. If the weight of an edge is higher than the threshold, the edge is retained.

Step 2 Pruning of the Gene Regulatory Network

In order to improve the false-positive rate, the TDCVSS model is utilized to select the regulatory factors of each target gene. According to the initial network, the optimal time factor vector, and gene expression data, the optimal TDCVSS model of each target gene is found by CVRAT and CVDE. If a regulatory factor is not included in the TDCVSS, this regulatory factor could not regulate the target gene. With such an approach, some false-positive regulatory relationships could be deleted in order to prune the network.

## Experiments

In this part, two real gene regulatory networks from *E. coli* and *Saccharomyces cerevisiae* are utilized. The true-positive rate (TPR), false-positive rate (FPR), positive predictive value (PPV), accuracy (ACC), and F-score are utilized to evaluate the performance of our method. In order to test our method well, the TDCVSS model is utilized to infer two real GRNs without the TDCC algorithm. Some classical GRN inference methods such as DBN (Perrin et al., 2003) [MMHO-DBN (Hu et al., 2020), DBN-ZC (Zou and Conzen, 2005) and DBmcmc (Husmeier, 2003)], RNN (Xu et al., 2007; Kordmahalleh et al., 2017), ODE (Chen et al., 2011), time-delayed methods [TDARACNE (Zoppoli et al., 2010), and TDLASSO (Mundra et al., 2013)] are also utilized.

### SOS Repair Network

**FIGURE 5:**
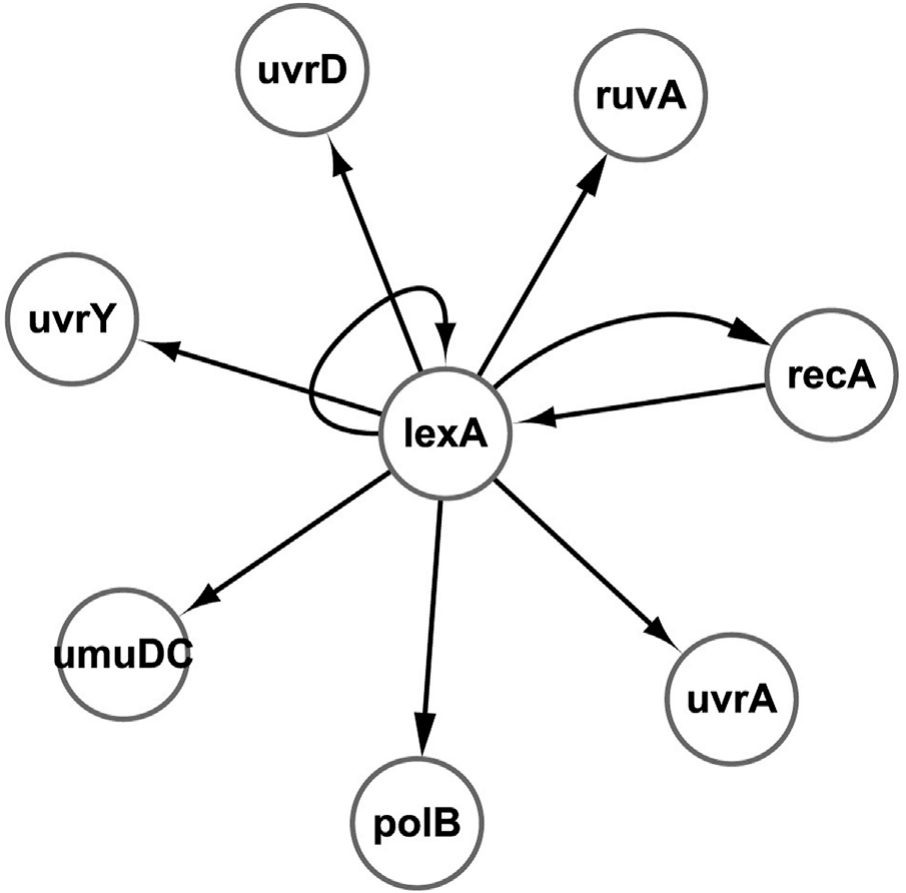
SOS DNA repair network of *E. coli*.

The first real biological gene expression data were derived from the SOS (Save Our Souls) DNA repair system. SOS DNA repair is a kind of DNA repair method induced by the serious damage of DNA and the cell in a crisis state under the action of a variety of enzymes, in order to maintain the integrity of the genome. The SOS reaction in DNA of *E. coli* is controlled by recA→lexA , and the network structure is depicted in Figure 5, which contains eight genes: uvrD, lexA, umuD, recA, uvrA, uvrY, ruvA, and polB (Ronen et al., 2002).

**FIGURE 6:**
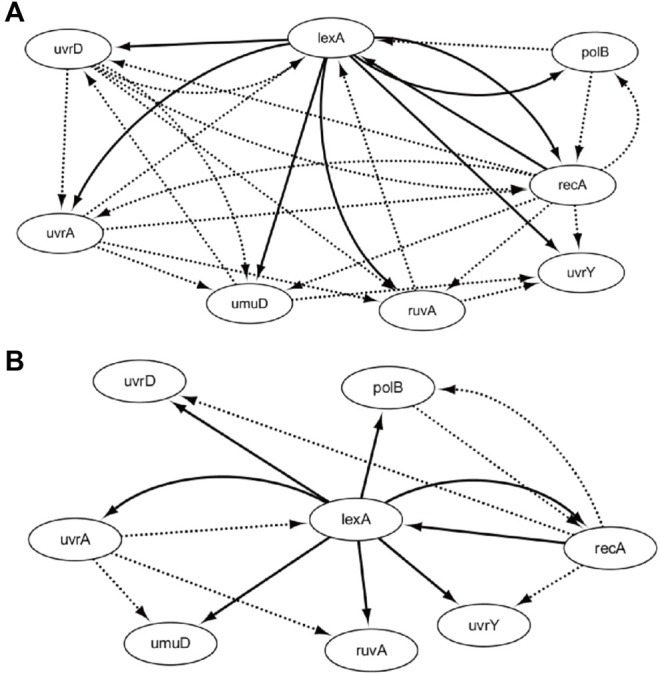
Network by CVTDSS **(A)** and the network by TDCC + CVTDSS **(B)**.

According to the gene expression levels, the time-delayed correlation coefficient is utilized to construct the network topology, which contains 29 regulatory relationships. Through our proposed evolutionary algorithm, the corresponding optimal CVTDSS models of eight target genes have been obtained in order to reduce the false-positive regulations and prune the network structures, which are described in Eq. 3. The final SOS repair network is obtained in Figure 6B, according to our proposed method. Figure 6A is the network obtained by CVTDSS without TDCC. The solid lines represent true-positive regulations, while dotted lines show the false-positive relationships. Comparing Figures 6A,B, it could be seen clearly that our proposed method infer less false-positive regulations.

$$\begin{array}{l} \frac{duvrD}{dt}=\left(-0.7775+1.909\;i\right)lexA_{t-1}^{2.7239}recA_{t}^{1.2357}-\left(-1.539+1.846\;i\right)lexA_{t-1}^{-1.586}recA_{t}^{-1.056},\\ \frac{dlexA}{dt}=\left(10.3037-46.2997\;i\right))uvrA_{t}^{1.4621}-\left(-7.1335-45.6055\;i\right)recA_{t}^{0.1352}uvrA_{t}^{3.0535},\\ \frac{dumuD}{dt}=\left(1.3215-0.6353\;i\right)uvrA_{t}^{-3.0207}-\left(0.3693-1.1433\;i\right)lexA_{t}^{-1.3984}uvrA_{t}^{0.7126},\\ \frac{drecA}{dt}=\left(19.3402+49.7278\;i\right)polB_{t}^{5.6735}-\left(3.7607+52.0372\;i\right)lexA_{t}^{2.7847},\\ \frac{duvrA}{dt}=\;\left(28.6246+20.8885\;i\right)lexA_{t}^{2.3048}-\left(-7.9147+17.355\;i\right)lexA_{t}^{3.2171},\\ \frac{duvrY}{dt}=\left(-1.0228+0.5707\;i\right)lexA_{t-2}^{-1.8106}recA_{t}^{-1.169}-\left(-0.5252+0.9496\;i\right)lexA_{t-2}^{1.9715},\\ \frac{druvA}{dt}=\left(0.4443+0.8445\;i\right)lexA_{t}^{0.8982}-\left(1.8336+0.9561\;i\right)lexA_{t}^{0.5566}uvrA_{t}^{-0.6937},\\ \frac{dpolB}{dt}=\left(0.4988+0.9654\;i\right)lexA_{t}^{0.1971}recA_{t}^{-1.0292}-\left(0.2515+1.0799\;i\right)recA_{t}^{0.9558}. \end{array}$$ (3)

DBN, RNN, S-system, and ODE are also utilized to infer the SOS repair network, and the results are listed in Table 1. From Table, we can figure out that in terms of TPR, our method and CVSS have the same performance, which is 59.99% higher than the S-system and RNN, 100% higher than DBN, and 33.27% higher than ODEs, which reveal that our method could infer more real regulations. In terms of FPR, RNN has the best performance. Our method only performs better than S-system, ODE, and CVSS, which show that the network obtained by our method does not have the least false-positive edges, but it has fewer wrong regulations. In terms of PPV, RNN has the highest performance, while our method has the second best performance. In terms of ACC, our method is 31.2% higher than the S-system, 16% higher than DBN, 8.4% higher than ODE, and 33.3% higher than CVSS. The results of F-score show that our method performs best as a whole.

**TABLE 1:** Performance comparison of six methods for the SOS network.

	TPR	FPR	PPV	ACC	F-Score
S-system	0.5556	0.20833	0.3333	0.6667	0.41667
DBN	0.4444	0.10417	0.44444	0.75439	0.44444
RNN	0.55556	0.041667	0.71429	0.80702	0.625
ODEs	0.6667	0.3125	0.28571	0.57895	0.4
TDCVSS	0.88889	0.3818	0.27586	0.65625	0.42105
Our method	0.88889	0.12723	0.5333	0.875	0.6667

### IRMA Network

**FIGURE 7:**
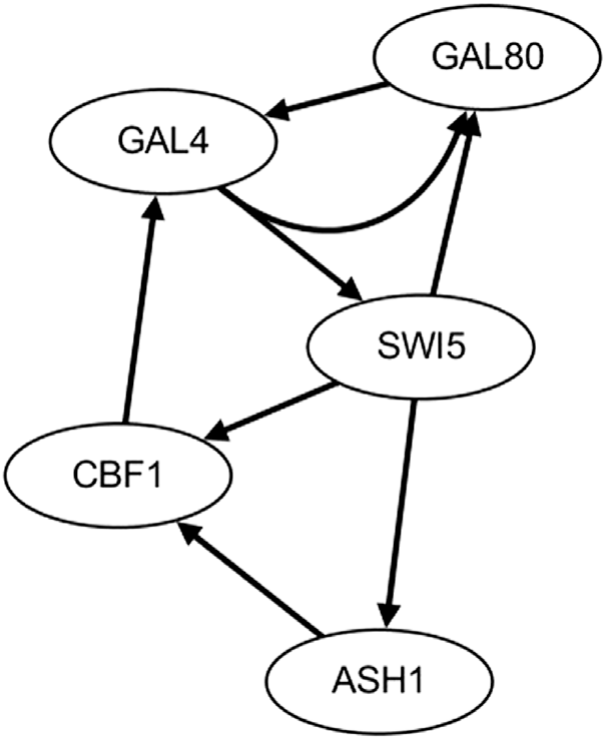
IRMA network.

The second real gene expression data are from the IRMA network, which is extracted from the switch process of galactose and glucose in *Saccharomyces cerevisiae*. According to the on and off of galactose creation, two kinds of gene expression datasets (on dataset and off dataset) are collected (Cantone et al., 2009). The real IRMA network is depicted in Figure 7.

**FIGURE 8:**
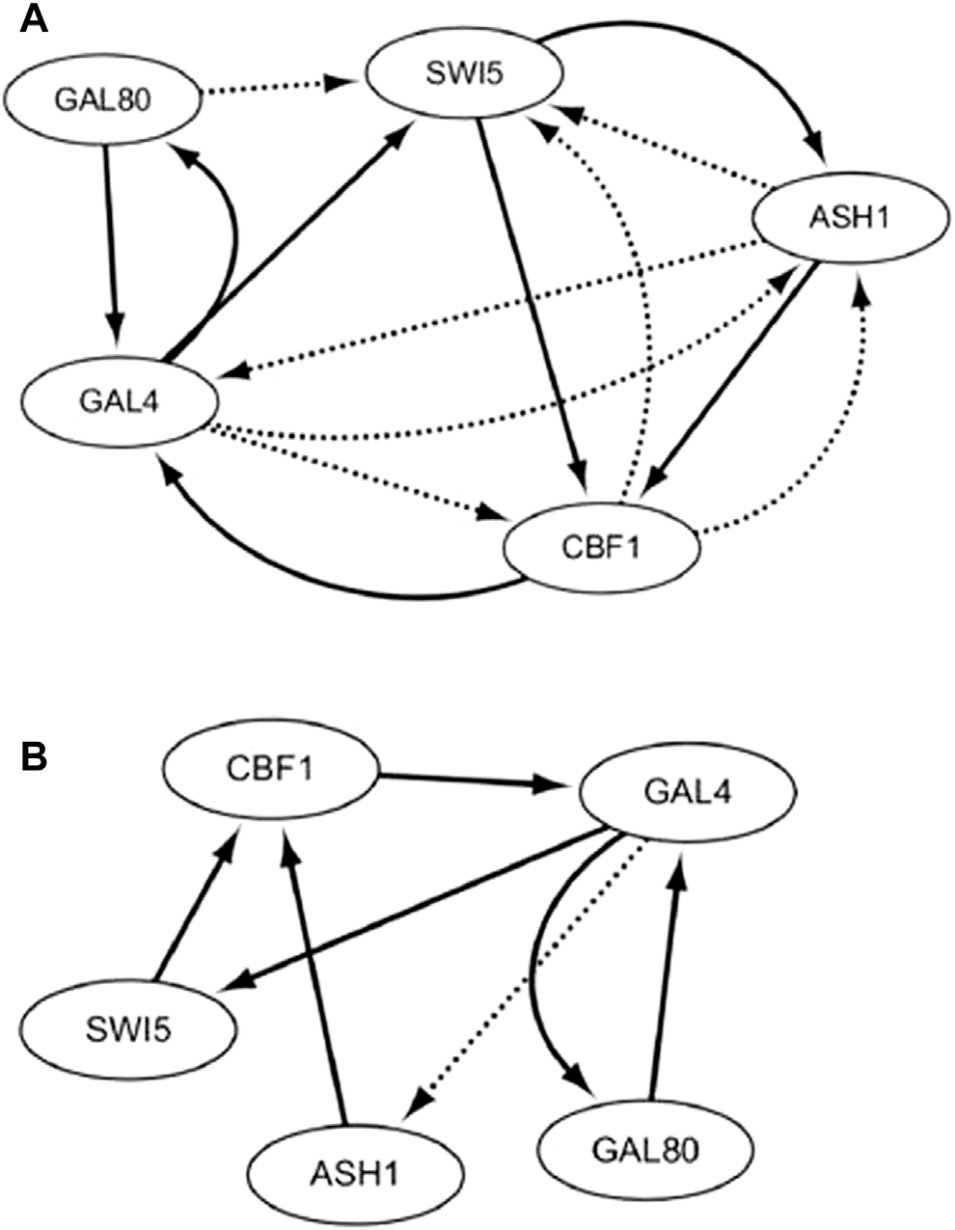
IRMA network by CVTDSS **(A)** and the IRMA network by TDCC + CVTDSS **(B)** with the on dataset.

With the on dataset, the TDCC could select 13 potential regulations for the initial IRMA network. According to the initial network and on dataset, five optimal CVTDSS models are found for five target genes (Eq. 4), which are utilized to determine the regulations of target genes and construct the final network (Figure 8B). The network inferred only by CVTDSS with the on dataset is depicted in Figure 8A. The solid lines represent true-positive regulations, while dotted lines show the false-positive relationships. Comparing two networks, it could be seen that our method could infer six real regulations, while CVSS can gain seven real relationships. But our method could infer less false-positive regulators and obtain a more accurate network.

$$\begin{array}{l} \frac{dCBF1}{dt}=\left(14.3404-4.3662\;i\right)SWI5_{t}^{-0.4442}-\left(18.5262+0.3632\;i\right)ASH1_{t}^{2.3205},\\ \frac{dGAL4}{dt}=\left(20.1982+23.7081\;i\right)GAL80_{t}^{-1.5041}-\left(27.7833+24.3643\;i\right)CBF1_{t-2}^{0.02835}\\ GAL80_{t}^{3.6337},\\ \frac{dSWI5}{dt}=\left(17.9446+14.6087\;i\right)GAL4_{t-1}^{-0.7849}-\left(17.152+16.076\;i\right)GAL4_{t-1}^{2.4668},\\ \frac{dGAL80}{dt}=\left(22.8761-19.8769\;i\right)GAL4_{t}^{2.6458}-\left(18.7878-18.4917\;i\right)GAL4_{t}^{-1.3359},\\ \frac{dASH1}{dt}=\left(17.8996+6.0684\;i\right)GAL4_{t-1}^{-0.0102}\left(5.355175+3.733905\;i\right)GAL4_{t-1}^{-3.7122}. \end{array}$$ (4)

HRNN, MMHO-DBN, TDARACNE, TDLASSO, DBmcmc, and DBN-ZC are also utilized to infer the IRMA network with the on dataset. Also, the results are described in Table 2. In terms of TPR, the CVSS model obtains the best performance, which proves that CVSS could infer more true-positive edges. Our method infers one edge less than CVSS, which may be because the TDCC method deletes this edge in the previous step. In terms of FPR, MMHO-DBN could gain zero, which reveals that the network inferred has no false-positive relationships. Our method could infer one false-positive edge and gain the second best FPR performance, which is 0.05882. In terms of ACC, our method could gain the highest accuracy, which shows that our method could infer more true-false edges and less false-positive edges. As a whole, our method has the highest F-score values. From the table, we could see that our method performs better than classical methods (HRNN, MMHO-DBN, DBmcmc, and DBN-ZC) and time-delayed methods (TDCVSS, TDARACNE, and TDLASSO).

**TABLE 2:** Performance comparison of eight methods for IRMA network inference with the on dataset.

Method	TPR	FPR	PPV	ACC	F-Score
Our method	0.75	0.05882	0.857,143	0.88	0.8
TDCVSS	0.875	0.411,765	0.5	0.68	0.636,364
HRNN	0.75	0.176,471	0.667	0.8	0.706,069
MMHO-DBN	0.5	0	1	0.84	0.666,667
TDARACNE	0.625	0.117,647	0.7142	0.8	0.666,629
TDLASSO	0.25	0.176,471	0.4	0.64	0.307,692
DBmcmc	0.25	0.117,647	0.5	0.68	0.333,333
DBN-ZC	0.375	0.117,647	0.6	0.72	0.461,538

**FIGURE 9:**
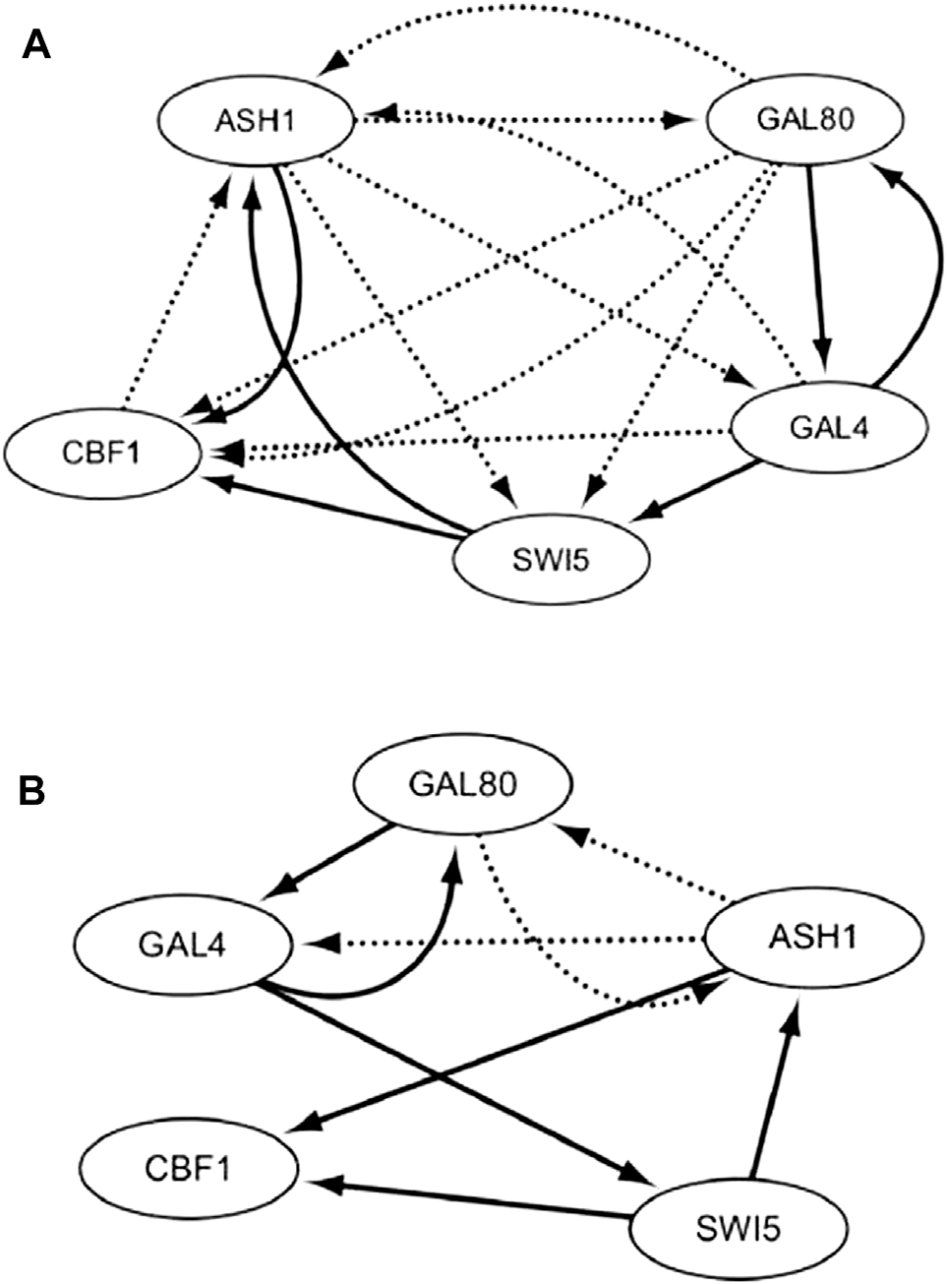
IRMA network by CVTDSS **(A)** and the IRMA network by TDCC + CVTDSS **(B)** with the off dataset.

With the off dataset, the TDCC could select 15 potential regulations for the initial IRMA network. According to the initial network and off dataset, five optimal CVTDSS models are found for five target genes (Eq. 5), which are utilized to determine the regulations of target genes and construct the final network (Figure 9B). The network inferred only by TDCVSS with the off dataset is depicted in Figure 9A. The solid lines represent true-positive regulations, while dotted lines show the false-positive relationships. Compared with two networks, our method and TDCVSS could infer the same number of true-positive edges, which is six. But our method could infer less false-positive regulators.

$$\begin{array}{l} \frac{dCBF1}{dt}=\left(10.5168+14.8629\;i\right)SWI5_{t-1}^{-2.172}-\left(4.345+5.378\;i\right)SWI5_{t-1}^{7.2487}ASH1_{t-1}^{-5.6179},\\ \frac{dGAL4}{dt}=\left(18.1621+3.7553\;i\right)ASH1_{t}^{-0.2432}-\left(17.870989-0.358178\;i\right)GAL80_{t}^{0.9609}ASH1_{t}^{-0.0552},\\ \frac{dSWI5}{dt}=\left(24.728+5.6469\;i\right)GAL4_{t-1}^{-4.489}-\left(27.7649+8.0166\;i\right)GAL4_{t-1}^{5.759},\\ \frac{dGAL80}{dt}=\left(4.1949-0.9859\;i\right)GAL4_{t}^{0.4187}ASH1_{t}^{1.782}-\left(5.268426-0.6605\;i\right)GAL4_{t}^{2.1168}ASH1_{t}^{-1.3287},\\ \frac{dASH1}{dt}=\left(2.8048-9.2581\;i\right)GAL80_{t}^{2.3158}-\left(9.133108-7.3237\;i\right)SWI5_{t}^{-2.1711}GAL80_{t}^{4.2043}. \end{array}$$ (5)

With the off dataset MMHO-DBN, TDARACNE, TDLASSO, and DBmcmc are also utilized to infer the IRMA network. The results are listed in Table 3. In terms of TPR, our method and the TDCVSS model can obtain the best performance, which is 0.75. Compared with other methods, these two methods could infer more true-positive edges. In terms of FPR, MMHO-DBN and TDARACNE have better performance, which reveals that the networks inferred by the two methods have fewer false-positive relationships than other methods. Our method and TDLASSO could gain the second best FPR performance, which is 0.176,471. TDCVSS has the worst FPR value. In terms of PPV, our method and MMHO-DBN obtain the best performance, which is 77.79% higher than TDCVSS, 33.34% higher than TDARACNE, 166.7% higher than TDLASSO, and 292.2% higher than DBmcmc. In terms of ACC and F-score, our method could gain the best performance, which shows that our method could infer more true-false edges and fewer false-positive edges than other methods as a whole.

**TABLE 3:** Performance comparison of six methods for IRMA network inference with the off dataset.

Method	TPR	FPR	PPV	ACC	F-Score
Our method	0.75	0.176,471	0.6667	0.8	0.705,901
TDCVSS	0.75	0.588,235	0.375	0.52	0.5
MMHO-DBN	0.25	0.058824	0.6667	0.72	0.363,641
TDARACNE	0.125	0.058824	0.5	0.68	0.2
TDLASSO	0.125	0.176,471	0.25	0.6	0.166,667
DBmcmc	0.12	0.294,118	0.17	0.52	0.14069

## Conclusion

In order to improve the accuracy of time-delayed GRN inference, a novel GRN inference method is proposed. In our method, the time-delayed correlation coefficient algorithm is first utilized to construct the TDCC matrix and the optimal time delay vector between genes. According to the TDCC matrix, the initial gene regulatory network topology is inferred. In order to improve the false-positive rate of GRN obtained, the time-delayed complex-valued S-system model is proposed to identify the regulations of each target gene, which could delete many false-positive relationships. When tested on two real gene expression datasets from *E. coli* and *S. cerevisiae* gene networks, in terms of F1, our method could make the 13.3–406.5% improvements, which show that our method as a whole has better performances than DBN (MMHO-DBN, DBN-ZC, and DBmcmc), RNN, ODE, and time-delayed methods (TDARACNE and TDLASSO).

From the results, it could be seen that our method could infer more true-positive regulations and fewer false-positive relationships than other classical GRN inference methods. However, each gene needs to identify the optimal CVTDSS model through an evolutionary algorithm, so the runtime of the proposed algorithm may be high. In future research, the parallel computing framework is planned to improve the time efficiency of the algorithm.

## Data Availability

The original contributions presented in the study are included in the article/Supplementary Material, further inquiries can be directed to the corresponding author.

## References

[B1] Abduallah Y. Wang J. T. L. “A Time-Delayed Information-Theoretic Approach to the Reverse Engineering of Gene Regulatory Networks Using Apache Spark,” in 2017 IEEE 15th Intl Conf on Dependable, Autonomic and Secure Computing, 15th Intl Conf on Pervasive Intelligence and Computing, 3rd Intl Conf on Big Data Intelligence and Computing and Cyber Science and Technology Congress(DASC/PiCom/DataCom/CyberSciTech), Orlando, FL, USA, 6-10 Nov. 2017, 1106–1113. 10.1109/dasc-picom-datacom-cyberscitec.2017.179

[B2] Bakbak P. Ö. Peker M. (2020). Classification of Sonar echo Signals in Their Reduced Sparse Forms Using Complex-Valued Wavelet Neural Network. Neural Comput. Applic 32 (7), 2231–2241. 10.1007/s00521-018-3920-4

[B3] Bracken C. P. Scott H. S. Goodall G. J. (2016). A Network-Biology Perspective of microRNA Function and Dysfunction in Cancer. Nat. Rev. Genet. 17 (12), 719–732. 10.1038/nrg.2016.134 27795564

[B4] Cantone I. Marucci L. Iorio F. Ricci M. A. Belcastro V. Bansal M. (2009). A Yeast Synthetic Network for *In Vivo* Assessment of Reverse-Engineering and Modeling Approaches. Cell 137, 172–181. 10.1016/j.cell.2009.01.055 19327819

[B5] Chen S. Mclaughlin S. Mulgrew B. (1994). Complex-valued Radial Basic Function Network, Part I: Network Architecture and Learning Algorithms. Signal. Process. 35 (1), 19–31. 10.1016/0165-1684(94)90187-2

[B6] Chen Y. Yang B. Meng Q. Zhao Y. Abraham A. (2011). Time-series Forecasting Using a System of Ordinary Differential Equations. Inf. Sci. 181 (1), 106–114. 10.1016/j.ins.2010.09.006

[B7] Chowdhury A. R. Chetty M. Vinh N. X. (2013). Incorporating Time-Delays in S-System Model for Reverse Engineering Genetic Networks. BMC Bioinformatics 14, 196. 10.1186/1471-2105-14-196 23777625 PMC3839642

[B8] Chowdhury A. R. Chetty M. Vinh N. X. (2013). “Reverse Engineering Genetic Networks with Time-Delayed S-System Model and Pearson Correlation Coefficient,” in Neural Information Processing. ICONIP 2013. Lecture Notes in Computer Science. Editors Lee M. Hirose A. Hou Z. G. Kil R. M. (Berlin, Heidelberg: Springer), Vol. 8227, 624–631. 10.1007/978-3-642-42042-9_77

[B9] Das S. Suganthan P. N. (2011). Differential Evolution: A Survey of the State-Of-The-Art. IEEE Trans. Evol. Computat. 15 (1), 4–31. 10.1109/tevc.2010.2059031

[B10] Fink O. Zio E. Weidmann U. (2014). Predicting Component Reliability and Level of Degradation with Complex-Valued Neural Networks. Reliability Eng. Syst. Saf. 121, 198–206. 10.1016/j.ress.2013.08.004

[B11] Goh S. L. Chen M. Popović D. H. Aihara K. Obradovic D. Mandic D. P. (2006). Complex-valued Forecasting of Wind Profile. Renew. Energ. 31 (11), 1733–1750. 10.1016/j.renene.2005.07.006

[B12] Gonzalez O. R. Küper C. Jung K. Naval P. C. Mendoza E. (2007). Parameter Estimation Using Simulated Annealing for S-System Models of Biochemical Networks. Bioinformatics 23 (4), 480–486. 10.1093/bioinformatics/btl522 17038344

[B13] Hernández-Prieto M. A. Semeniuk T. A. Futschik M. E. (2014). Toward a Systems-Level Understanding of Gene Regulatory, Protein Interaction, and Metabolic Networks in Cyanobacteria. Front. Genet. 5, 191. 10.3389/fgene.2014.00191 25071821 PMC4079066

[B14] Hu H. Guan Q. Chen S. Ji Z. Lin Y. (2020). Detection and Recognition for Life State of Cell Cancer Using Two-Stage cascade CNNs. Ieee/acm Trans. Comput. Biol. Bioinf. 17 (3), 887–898. 10.1109/TCBB.2017.2780842 29990223

[B15] Husmeier D. (2003). Sensitivity and Specificity of Inferring Genetic Regulatory Interactions from Microarray Experiments with Dynamic Bayesian Networks. Bioinformatics 19 (17), 2271–2282. 10.1093/bioinformatics/btg313 14630656

[B16] Iwata M. Sriyudthsak K. Hirai M. Y. Shiraishi F. (2014). Estimation of Kinetic Parameters in an S-System Equation Model for a Metabolic Reaction System Using the Newton-Raphson Method. Math. Biosciences 248, 11–21. 10.1016/j.mbs.2013.11.002 24291302

[B17] Ji Z. Yan K. Li W. Hu H. Zhu X. (2017). Mathematical and Computational Modeling in Complex Biological Systems. Biomed. Research International 2017, 1–16. 10.1155/2017/5958321 PMC5366773 28386558

[B18] Kordmahalleh M. M. Sefidmazgi M. G. Harrison S. H. Homaifar A. (2017). Identifying Time-Delayed Gene Regulatory Networks via an Evolvable Hierarchical Recurrent Neural Network. Biodata Mining 10 (1), 29. 10.1186/s13040-017-0146-4 28785315 PMC5543747

[B19] Li P. Gong P. Li H. Perkins E. J. Wang N. Zhang C. (2014). Gene Regulatory Network Inference and Validation Using Relative Change Ratio Analysis and Time-Delayed Dynamic Bayesian Network. J. Bioinform Sys Biol. 2014, 12. 10.1186/s13637-014-0012-3 PMC5270498 28194162

[B20] Liu C. Chyr J. Zhao W. Xu Y. Ji Z. Tan H. (2018). Genome-Wide Association and Mechanistic Studies Indicate that Immune Response Contributes to Alzheimer's Disease Development. Front. Genet. 9, 410. 10.3389/fgene.2018.00410 30319691 PMC6166008

[B21] Liu P.-K. Wang F.-S. (2008). Inference of Biochemical Network Models in S-System Using Multiobjective Optimization Approach. Bioinformatics 24 (8), 1085–1092. 10.1093/bioinformatics/btn075 18321886

[B22] Lo L.-Y. Leung K.-S. Lee K.-H. (2015). Inferring Time-Delayed Causal Gene Network Using Time-Series Expression Data. Ieee/acm Trans. Comput. Biol. Bioinf. 12 (5), 1169–1182. 10.1109/tcbb.2015.2394442 26451828

[B23] Ma'ayan A. (2009). Insights into the Organization of Biochemical Regulatory Networks Using Graph Theory Analyses. J. Biol. Chem. 284 (9), 5451–5455. 10.1074/jbc.r800056200 18940806 PMC2645810

[B24] Miyawaki-Kuwakado A. Komori S. Shiraishi F. (2020). A Promising Method for Calculating True Steady-State Metabolite Concentrations in Large-Scale Metabolic Reaction Network Models. Ieee/acm Trans. Comput. Biol. Bioinf. 17 (1), 27–36. 10.1109/tcbb.2018.2853724 30004883

[B25] Mundra P. A. Zheng J. Niranjan M. Welsch R. E. Rajapakse J. C. (2013). “Inferring Time-Delayed Gene Regulatory Networks Using Cross-Correlation and Sparse Regression,” in Bioinformatics Research and Applications. ISBRA 2013. Lecture Notes in Computer Science. Editors Cai Z. Eulenstein O. Janies D. Schwartz D. (Berlin, Heidelberg: Springer), Vol. 7875, 64–75. 10.1007/978-3-642-38036-5_10

[B26] Parmar K. Blyuss K. B. Kyrychko Y. N. Hogan S. J. (2015). Time-Delayed Models of Gene Regulatory Networks. Comput. Math. Methods Med. 2015, 1–16. 10.1155/2015/347273 PMC4632181 26576197

[B27] Perrin B.-E. Ralaivola L. Mazurie A. Bottani S. Mallet J. d'Alche-Buc F. (2003). Gene Networks Inference Using Dynamic Bayesian Networks. Bioinformatics 19, ii138–ii148. 10.1093/bioinformatics/btg1071 14534183

[B28] Quach M. Brunel N. d'Alche-Buc F. (2007). Estimating Parameters and Hidden Variables in Non-linear State-Space Models Based on ODEs for Biological Networks Inference. Bioinformatics 23 (23), 3209–3216. 10.1093/bioinformatics/btm510 18042557

[B29] Rashid S. Saraswathi S. Kloczkowski A. Sundaram S. Kolinski A. (2016). Protein Secondary Structure Prediction Using a Small Training Set (Compact Model) Combined with a Complex-Valued Neural Network Approach. BMC Bioinformatics 17, 362. 10.1186/s12859-016-1209-0 27618812 PMC5020447

[B30] Ronen M. Rosenberg R. Shraiman B. I. Alon U. (2002). Assigning Numbers to the Arrows: Parameterizing a Gene Regulation Network by Using Accurate Expression Kinetics. Proc. Natl. Acad. Sci. U.S.A. 99, 10555–10560. 10.1073/pnas.152046799 12145321 PMC124972

[B31] Rupaimoole R. Calin G. A. Lopez-Berestein G. Sood A. K. (2016). miRNA Deregulation in Cancer Cells and the Tumor Microenvironment. Cancer Discov. 6, 235–246. 10.1158/2159-8290.cd-15-0893 26865249 PMC4783232

[B32] Savitha R. Suresh S. Sundararajan N. (2012). Fast Learning Circular Complex-Valued Extreme Learning Machine (CC-ELM) for Real-Valued Classification Problems. Inf. Sci. 187, 277–290. 10.1016/j.ins.2011.11.003

[B33] Sefidmazgi A. G. Ahmadi-Abkenari F. Mirroshandel S. A. “Correlation Analysis as a Dependency Measures for Inferring of Time-Lagged Gene Regulatory Network,” in 2016 Eighth International Conference on Information and Knowledge Technology (IKT), Hamedan, Iran, 7-8 Sept. 2016, 6–11. 10.1109/ikt.2016.7777761

[B34] Tesniere A. Schlemmer F. Boige V. Kepp O. Martins I. Ghiringhelli F. (2010). Immunogenic Death of colon Cancer Cells Treated with Oxaliplatin. Oncogene 29 (4), 482–491. 10.1038/onc.2009.356 19881547

[B35] Thomas R. K. Baker A. C. Debiasi R. M. Winckler W. Laframboise T. Lin W. M. (2007). High-throughput Oncogene Mutation Profiling in Human Cancer. Nat. Genet. 39 (3), 347–351. 10.1038/ng1975 17293865

[B36] Wang G. Yang Z. Turcotte M. (2020). Dynamic Analysis of the Time-Delayed Genetic Regulatory Network between Two Auto-Regulated and Mutually Inhibitory Genes. Bull. Math. Biol. 82, 46. 10.1007/s11538-020-00722-1 32236721

[B37] Wang H. Dougherty E. Qian L. (2010). Inference of Gene Regulatory Networks Using S-System: A Unified Approach. Iet Syst. Biol. 4 (2), 145–156. 10.1049/iet-syb.2008.0175 20232994

[B38] Xu R. Wunsch D. C. Frank R. L. (2007). Inference of Genetic Regulatory Networks with Recurrent Neural Network Models Using Particle Swarm Optimization. Ieee/acm Trans. Comput. Biol. Bioinf. 4 (4), 681–692. 10.1109/tcbb.2007.1057 17975278

[B39] Yang B. Bao W. Chen Y. (2020). Time Series Prediction Based on Complex-Valued S-System Model. Complexity 2020, 1–13. 10.1155/2020/6393805

[B40] Yang B. Bao W. (2019). Complex-Valued Ordinary Differential Equation Modeling for Time Series Identification. IEEE ACCESS 7, 41033–41042. 10.1109/access.2019.2902958

[B41] Yu B. Xu J.-M. Li S. Chen C. Chen R.-X. Wang L. (2017). Inference of Time-Delayed Gene Regulatory Networks Based on Dynamic Bayesian Network Hybrid Learning Method. Oncotarget 8 (46), 80373–80392. 10.18632/oncotarget.21268 29113310 PMC5655205

[B42] Yuan M. Wang W. Wang Z. Luo X. Kurths J. (2021). Exponential Synchronization of Delayed Memristor-Based Uncertain Complex-Valued Neural Networks for Image Protection. IEEE Trans. Neural Netw. Learn. Syst. 32 (1), 151–165. 10.1109/tnnls.2020.2977614 32203028

[B43] Zhang P. Xia J.-H. Zhu J. Gao P. Tian Y.-J. Du M. (2018). High-throughput Screening of Prostate Cancer Risk Loci by Single Nucleotide Polymorphisms Sequencing. Nat. Commun. 9 (1), 2022. 10.1038/s41467-018-04451-x 29789573 PMC5964124

[B44] Zhao Y. Jiang M. Chen Y. (2016). Inferring Gene Regulatory Networks Using a Time-Delayed Mass Action Model. J. Bioinform. Comput. Biol. 14, 1650012. 10.1142/s0219720016500128 27093908

[B45] Zoppoli P. Morganella S. Ceccarelli M. (2010). TimeDelay-ARACNE: Reverse Engineering of Gene Networks from Time-Course Data by an Information Theoretic Approach. BMC Bioinformatics 11, 154. 10.1186/1471-2105-11-154 20338053 PMC2862045

[B46] Zou M. Conzen S. D. (2005). A New Dynamic Bayesian Network (DBN) Approach for Identifying Gene Regulatory Networks from Time Course Microarray Data. Bioinformatics 21 (1), 71–79. 10.1093/bioinformatics/bth463 15308537

